# A questionnaire-based study to comprehensively assess the status quo of rare disease patients and care-givers in China

**DOI:** 10.1186/s13023-021-01954-7

**Published:** 2021-07-22

**Authors:** Xuefeng Li, Meiling Liu, Jinduan Lin, Bingzhe Li, Xiangyu Zhang, Shu Zhang, Zijuan Lu, Jianyong Zhang, Jincheng Zhou, Li Ou

**Affiliations:** 1 The Sixth Affiliated Hospital of Guangzhou Medical University, Qingyuan People’s Hospital, Qingyuan, People’s Republic of China; 2 State Key Laboratory of Respiratory Disease, Sino-French Hoffmann Institute, School of Basic Medical Sciences, Guangzhou Medical University, Guangzhou, 511436 People’s Republic of China; 3 Shenzhen Luohu People’s Hospital, The Third Affiliated Hospital of Shenzhen University, Shenzhen, 518001 People’s Republic of China; 4 School of Electrical and Computer Engineering, Oklahoma State University, Stillwater, OK 74078 USA; 5 School of Statistics, University of Minnesota, Minneapolis, MN 55455 USA; 6 Department of Oral Implantology, The Affiliated Stomatology Hospital of Kunming Medical University, Kunming, 650106 People’s Republic of China; 7 School of Humanities, Tongji University, Shanghai, 200092 People’s Republic of China; 8 Jinhaishiji, 333 Jichanglu, Panzhihua, 617000 Sichuan People’s Republic of China; 9 Center for Design and Analysis, Amgen Inc., Thousand Oaks, CA 91320 USA; 10 Gene Therapy Center, Department of Pediatrics, University of Minnesota, 5-174 MCB, 420 Washington Ave SE, Minneapolis, MN 55455 USA

**Keywords:** Rare disease, Orphan drug, Patient organizations, Questionnaire

## Abstract

**Background:**

There are over 16.8 million rare disease patients in China, representing a large community that should not be neglected. While the public lack the awareness of their existence and difficult status quo, for one reason that they exist as a rare and special group in our society, for another reason that all sectors of the community haven’t introduced and propagandized them suitably. However, as a special group with more difficulties in all aspects than normal healthy persons, they need enough care and love from us. To provide a basis for policy-makers to better understand the status quo of rare disease patients and care-givers in China and to devise some new policies to improve their quality of life, a comprehensive analysis of the status quo, unmet needs, difficulty caused by the rare disease is essential.

**Methods:**

A questionnaire-based online study of patients and care-givers (usually family members) was performed. The questionnaire was composed of 116 questions, such as the diagnosis process, treatment access, financial burden, views on patients’ organizations, and a series of standardized tests to assess the quality of their life, including the SF-36, PHQ-9, PHQ-15, GAD-7, and PSQI. To examine the influence of age, disease type, and relationship to patients on the scores in these tests, statistical analysis with a general linear model was conducted.

**Findings:**

A total of 1959 patients and care-givers participated in the survey, representing 104 rare diseases, such as lysosomal storage diseases, hemophilia, and muscular dystrophy diseases. The diagnosis was delayed for 1.4 ± 3.0 years, and patients experienced 1.6 ± 3.8 misdiagnoses between 3.2 ± 2.4 hospitals. The hospitals where diagnoses were made were highly concentrated in 10 large hospitals (43.8%) and 5 big cities (42.1%), indicating a significant inequality of medical resources. The disease often led to difficulty in social life, education, and employment, as well as financial burden that was seldom covered by medical insurance. A battery of standardized tests demonstrated poor health status, depression, somatization, anxiety, and sleeping issues among both patients and care-givers (*p* < 0.05). Statistical analysis of the questionnaire also showed that poor health, anxiety, depression, somatization, and sleeping problems were more prevalent in patients than in care-givers, and more prevalent in more severe diseases (e.g., hemophilia, Dravet) or undiagnosed than in other diseases.

**Interpretations:**

This study identified the lack of rare disease awareness and legislative support as the major challenge to rare diseases in China, and makes key recommendations for policy-makers, including legislating orphan drug act, raising rare disease awareness, providing sufficient and fair opportunities about education and employment, expanding the medical insurance coverage of treatments, and protecting rights in education and employment.

**Supplementary Information:**

The online version contains supplementary material available at 10.1186/s13023-021-01954-7.

## Introduction

A rare disease is any disease that affects a relatively small number of people. There is no universal definition of rare diseases, and definitions vary in different countries. In America, a rare disease is defined as a disease affecting less than 200,000 persons each year, while in Europe it is a disease which affects less than 5 in 10,000 persons [1]. Besides, Japan stipulates that rare diseases are those affecting less than 50,000 people [2], while Taiwan takes the incidence rate of less than 1/10,000 as the standard for rare diseases [3]. Because of the genetic information, living habits and environment of different ethnic groups are diverse, the prevalence rates of all kinds of diseases may also be different. Hence, a disease is defined as a rare disease in a race, while it may not fall within the definition range of rare disease in another race. Besides, as the population morbidity changes over time, whether certain disease belongs to the scope of rare diseases can also change. Although the definition and scope of rare diseases vary in different countries, their kinds are still various and affect many people all around the world. According to statistics, there are between 5000 and 8000 rare diseases [4], affecting a total of 400 million patients worldwide [5]. Together, rare diseases affect approximately 6–10% of the population and 3–4% of births [6], thus rare diseases pose a significant challenge to the healthcare systems. Approximately 80% of rare diseases are genetic, many of which are life-threatening [7]. Most rare disease patients are children, and 30% of patients die before the age of 5 years [8]. Delayed diagnosis and misdiagnosis are common, resulting in inappropriate treatment and poor outcomes [9]. Approximately 95% of rare diseases have no treatment options [8]. Even if treatments exist, the availability and affordability of treatment are often poor, especially in developing countries. Awareness and knowledge of rare diseases are often lacking, rendering many patients struggling to find adequate information. Consequently, the emotional, psychological, and financial impact of a rare disease on patients and care-givers is also considerable, which should be attached great importance to.

There is still no official definition of rare diseases in China; therefore, the total number of rare disease patients in China is unknown. It was previously estimated to be 16.8 million [10]. However, this may be a significant underestimate considering the population of 1.4 billion.

Since the count of rare disease patients in China is enormous and cannot be ignored, the Chinese government has realized to take measures to improve the situation and life quality of rare disease patients in recent years. Excitingly, five Chinese government departments jointly officially issued the first list of rare diseases of China on May 22, 2018 [11]. According to the actual situation of rare diseases in China and the learning from international experiences, the list which includes 121 rare diseases such as hemophilia, albinism and amyotrophic lateral sclerosis, is selected by authoritative experts in different fields in accordance with certain working procedures. To further promote measures to combat rare diseases, in February 2019, the National Health Commission of the People's Republic of China announced the establishment of the "National Network to Collaborate on Diagnosis and Treatment of Rare Diseases" and formally issued the Guidelines for the Diagnosis and Treatment of Rare Diseases (2019), which is the first national guidelines for the diagnosis and treatment of rare diseases [12, 13].

In fact, medical insurance plays an important role in relieving the financial burden of illness in a large population. Nowadays, basic health insurance in China consists of the Urban Employee Basic Medical Insurance, the New Rural Cooperative Medical Scheme and the Urban Resident Basic Medical Insurance [14]. Since the first revision of the National Essential Medicines List (NEML), composed of 307 essential medicines, was issued by government in 2009, the latest NEML (2018) has increased the drugs to 685, which contains a large number of drugs for general diseases [15]. Residents can demand reimbursement for medical expenses according to different types of medical insurance, although the cost of most rare diseases are excluded.

In order to improve the medical insurance coverage of rare diseases, some local governments and non-governmental organizations have focused on the local rare diseases and take efforts to promote the implement of relevant beneficial policies for several years. In 2012, Qingdao issued the Opinions on Establishing the Medical Assistance System for Urban Serious Diseases (Trial), which clearly included rare diseases into the medical security system for serious diseases for the first time. In 2016, Zhejiang Provincial Government issued the Notice on strengthening medical security for rare diseases, which initially established a rare disease security mechanism in Zhejiang. In 2016, the List of Major Rare Diseases in Shanghai (2016), released by Shanghai Municipal Health and Family Planning Commission, clearly took 56 rare diseases on board [16]. Based on different local policies of medical insurance for rare diseases, patients can ask for reimbursement of medical expenses to some extent.

In some degree, the central government and local government do have paid attention to improving the current situation of rare diseases in recent years. However, compared with general healthcare resources, there still lacks legislation or medical insurance coverage of treatments for rare diseases in China, resulting in low interest in orphan drug development by the pharmaceutical industry. The rare disease field is largely underdeveloped due to the lack of rare disease awareness, legislation, pharmaceutical development, and insurance coverage in China. It is essential to have an in-depth understanding of this field to provide a basis for legislation.

Based on above reasons and purposes, this study aims to comprehensively assess the status quo of patients and care-givers, including the diagnostic process, financial and social stress, psychological impact, and quality of life, as well as their perspectives on relevant issues. Key recommendations to support patients, motivate orphan drug development, and improve rare disease awareness, are made based on the questionnaire results. The results will provide the basis for considerations of policy-makers. This is the first large-scale study to assess the diagnosis process, treatment access, financial stress, physical and mental health, and viewpoints on policies of patients and care-givers of rare diseases in China.

## Materials and methods

### Ethics statement

The study was approved by the Institutional Ethics Committee of Guangzhou Medical University. By the assistance of Seven Pansy Rare Diseases Platform, we recruited the potential participants (patients or care-givers, 18 years old or above) to finish the online questionnaire via network link on ‘zhihu’ website. And the respondents were encouraged to invite their friends with rare diseases to complete the survey. Care-givers are family members who take care of the patients. Those who are not rare disease patients or care-givers were not included in the analysis. Only those who signed the informed consent participated in this study between March and May 2020.

The questionnaire was composed of 116 questions such as diagnosis process, treatment access, financial burden, views on patients’ organizations, and a series of standardized tests, general named Health-related quality of life (HRQOL), to assess the quality of their life, including the SF-36, PHQ-9, PHQ-15, GAD-7, and PSQI. The reasons why we choose HRQOL indicators are as followed. Firstly, Centers for Disease Control of America has defined Health-related quality of life (HRQOL) as “an individual’s or group’s perceived physical and mental health over time. HRQOL not only links with individual physical and mental health perceptions and other factors—including their health risks and conditions, functional status, social support, and socioeconomic status, but also related to community resources, conditions, policies, and practices that influence a population’s health perceptions and functional status [17]. Secondly, analyzing the surveillance data from HRQOL can contribute to identifying subgroups with relatively poor perceived health and assist on guiding interventions to improve their status and avoid more serious aftermaths [17]. Besides, study and publication of these HRQOL data can conduce to identify needs for health policies and legislation, facilitate to reasonably allocate resources, guide the formulation of strategic plans better, and monitor the effectiveness of broad community interventions. Thirdly, the population with rare diseases, also known as a special group with relatively poor perceived health, covers very low percentage of all human beings, but the overall number of rare disease patients is enormous and can’t be ignored. In order to know their quality of life better and facilitate to improve their living actuality, we used the HRQOL indicators in questionnaire, which could reflect their real conditions of physical and mental health in an authentic and effective way.

All of the questions were designed in Chinese. The data received were stored in excel, and the missing data in the questions of assessment instruments were performed whole abandonment, then we analyzed the complete data in classification statistically. For example, if any data were missing in the part of assessment instruments, these respondents’ whole responses in evaluating their quality of life would be also excluded.

All the participants acknowledged; (1) that they would participate in this study anonymously; (2) that they could decline to answer any of the questions; (3) that they could quit the study at any time; (4) that the results would be published in a scientific journal without seeking their approval of the manuscript; and (5) that they would not be paid for participating in this study.

### Assessment instruments

The Short Form (36) Health Survey is a 36-item, self-reported survey on the status of health [18]. SF-36 includes 8 domains: physical function (PF), role limitations due to physical problems (RP), bodily pain (BP), general health (GH), vitality (VT), social function (SF), role limitations due to emotional problems (RE), and mental health (MH). The score of each domain ranges from 0 to 100, and a low score indicates poor health. The Pittsburgh Sleep Quality Index (PSQI) is a screening tool for sleeping difficulty. PSQI consists of 19 items that can be categorized into 7 domains, including subjective sleep quality (SQ), sleep latency (SL), sleep duration (SD), habitual sleep efficiency (SE), sleep disturbance (Dis), sleep medication use (SM), and daytime dysfunction (DD) [19]. The total score of PSQI ranges from 0 to 21, and a high score indicates poor sleep quality. Patient Health Questionnaire-9 (PHQ-9) is a 9-item screening tool for depression [20]. PHQ-9 measures the frequency of depression symptoms over the last 2 weeks. The total score of PHQ-9 ranges from 0 to 27, and scores of ≥ 5, ≥ 10, and ≥ 15, represent mild, moderate, and severe depression. PHQ-15 is a screening tool for somatization [21]. PHQ-15 measures the severity of individual somatic symptoms during the past 4 weeks. The total score of PHQ-15 ranges from 0 to 30, and scores of ≥ 5, ≥ 10, ≥ 15 represent mild, moderate, and severe somatization, respectively. Generalized Anxiety Disorder Scale (GAD-7) is a screening tool for anxiety [22]. Each item describes one of the typical anxiety symptoms and measures the frequency of each symptom over the past 2 weeks. The reliability and validity of the Chinese version of SF-36, PSQI, PHQ-9, PHQ-15, and GAD-7 have been confirmed [23–27].

### Data analysis

The Statistical Package for the Social Sciences (SPSS 20.0) was used to analyze data, and a *p* value < 0.05 (two-tailed tests) denotes statistical significance. The scores of the SF-36, PHQ-9, PHQ-15, GAD-7, and PSQI largely met normal distribution, therefore, a general linear model was applied. For SF-36, PHQ-9, PHQ-15, GAD-7, and PSQI, only those who completed all questions of each test were included in the quantitative analysis. Mean ± standard deviations and 95% confidence interval (CI) were used to describe continuous outcomes.

## Results

### Demographic information

**Fig. 1:**
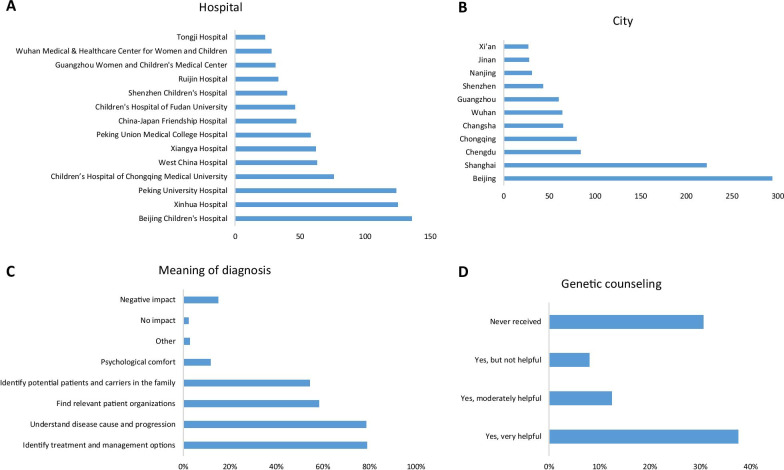
Information about diagnosis. **a** A list of hospitals where the most patients in this study were diagnosed. **b** A list of cities where the most patients in this study were diagnosed. **c** The meaning of diagnosis to patients and care-givers. **d** The information about genetic counseling

A total of 1959 individuals signed informed consent and participated in completing the questionnaire. The demographic information of participants is listed in Table 1. These participants represent 104 rare diseases and those diseases remaining undiagnosed after repeatedly visiting the hospital. The average onset age is 36.8 ± 85.1 months, while the average diagnosis age is 56.5 ± 110.0 months (95% CI 51.6–61.4). The diagnosis was delayed for 16.2 ± 35.4 months (95% CI 14.6–17.8). Interestingly, 497 patients (26.1%) were diagnosed through newborn screening. When patients diagnosed through newborn screening were excluded, the diagnosis was delayed for 24.3 ± 41.8 months (95% CI 22.1–26.5), with the onset age being 48.6 ± 95.0 months (95% CI 43.6–53.6), and diagnosis age being 77.9 ± 122.7 months (95% CI 71.5–84.3). During the diagnosis odyssey, patients visited 3.2 ± 2.4 hospitals (95% CI 3.1–3.3), and experienced 1.6 ± 3.8 misdiagnoses (95% CI 1.4–1.8). A total of 1770 participants provided information about 139 hospitals where the diagnoses were made. The hospitals that diagnosed more than 20 participants of this study were listed in Fig. 1a. The top 10 hospitals on the list diagnosed 777 patients (43.8% of total), and they were all located in big cities (Fig. 1b). The top 5 cities diagnosed a combined total of 745 patients (42.1%). There is a clear inequality of medical resources in China.

**Table 1:** Demographic information of participants of the questionnaire for patients and care-givers

Relationship	Patients (n = 340, 17.4%)
	Care-givers (n = 1599, 81.6%)
Gender	Male (n = 575, 29.6%)
	Female (n = 1369, 70.4%)
Age	Respondents (33.9 ± 7.4 years)
	Patients (10.6 ± 12.6 years)
	Care-givers (34.4 ± 6.7 years)
Locations	Shandong (n = 164)
	Sichuan (n = 144)
	Hubei (n = 140)
	Henan (n = 134)
	Guangdong (n = 128)
	Hunan (n = 109)
	Beijing (n = 103)
	Other (n = 999)
Disease	CAH (n = 419)
	PKU (n = 246)
	Hemophilia (n = 243)
	Dravet (n = 153)
	DMD (158)
	Neurofibromatosis (n = 69)
	Pompe (n = 40)
	MPS (n = 71)
	AHC (n = 28)
	Addison' disease (n = 26)
	SCA (n = 38)
	Undiagnosed (n = 193)
	Other (n = 275)

Only provinces/districts with more than 100 participants were shown. Only diseases with more than 20 participants were shown. Mean ± standard deviations

As shown in Fig. 1c, most respondents (79.0%) believed that diagnosis helped to figure out treatment and management options, while 1530 (78.6%) believed that diagnosis helped to understand the disease cause and progression. Interestingly, 290 individuals (14.9%) thought that diagnosis had negative impacts (depression, anxiety, guilt, denial, discrimination, and financial stress).

**Fig. 2:**
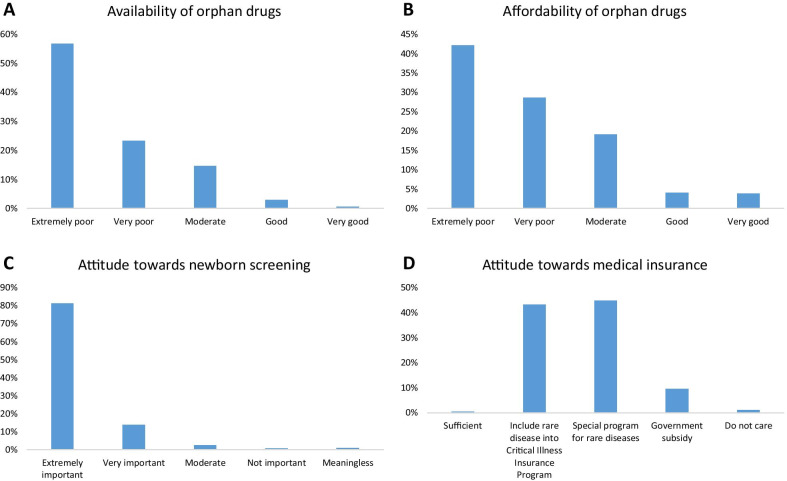
Attitude of patients and care-givers towards orphan drugs, newborn screening, and medical insurance. **a** The respondents’ viewpoints on availability of orphan drugs. **b** The respondents’ views on affordability of orphan drugs. **c** The respondents’ attitude towards newborn screening. **d** The opinions and suggestions of patients and care-givers about medical insurance

As shown in Fig. 1d, 687 of 1831 respondents (37.5%) had received genetic counseling and believed it to be helpful, 146 (8.0%) believed it to be not helpful, while 227 (12.4%) believed it to be moderately helpful. A total of 560 patients (30.6%) had never received genetic counseling. Most respondents (72.6%) believed that their doctors did not have sufficient information about rare diseases. Most respondents (82.1%) deemed newborn screening to be extremely important (Fig. 2c).

### Orphan drugs and medical cost

As shown in Fig. 2, most respondents (57.7%) rated the availability of orphan drugs as extremely poor, and 456 individuals (23.7%) rated it as poor. As to the affordability of orphan drugs, most respondents (72.3%) rated it as extremely poor (43.0%) or poor (29.3%). Most respondents (50.4%) had spent 5–50% of their annual family income on the medical cost of rare diseases, 526 respondents (27.7%) had spent 50–100% of income, and 231 (12.2%) had spent > 100% of income. These results indicate that rare diseases caused a significant financial burden.

As to the specific types of medical insurance, most respondents (59.6%) had Basic Medical Insurance Systems for Urban and Rural Residents, 404 (20.8%) had Medical/Social Insurance Program for Children, and 112 (5.8%) had no medical insurance. However, only 0.8% (16/1913) of the individuals had orphan drug costs fully covered by insurance, 50.2% individuals had partial coverage, while the medical cost of 48.1% individuals was not covered by insurance at all. Out of 1936 respondents, 877 (45.3%) recommended to implement special insurance program for rare diseases, 847 (43.8%) recommended to include rare diseases into Critical Illness Insurance Program, a national medical insurance program. Only 8 individuals (0.4%) believed that the current insurance system is sufficient. These results demonstrate that there lacks sufficient support from the medical insurance system.

### Difficulty in life, education, and employment

Patients and care-givers are also often disadvantaged in employment, education, and social life [28]. In this study, only 37.6% of respondents did not need any assistance, while the remaining 62.4% needed different levels of assistance. However, 83.0% of patients had no disability certificates, which are issued by the government to provide benefits and protection. Out of 1830 respondents, 725 (39.8%) found no available barrier-free facilities, and 490 (26.9%) found very few such facilities. Only 61 (3.4%) and 277 (15.2%) believed that available barrier-free facilities could completely or largely meet their needs, respectively. Most respondents (59.7%) had experienced difficulty in education. As to the main financial source of patients, 69.6% of respondents relied on the income of their family members, and only 215 (11.4%) relied on their own income. Only 88 (4.7%), 23 (1.2%) and 65 (3.5%) of patients had full-time, part-time jobs, or free-lancers, respectively. A total of 823 patients (43.8%) were children or retired, 549 (29.2%) were in college and continuing education, and 333 (17.7%) were unemployed. Most respondents (62.0%) believed that patients had experienced difficulty in finding jobs. Regarding the specific challenges in finding jobs, 340 out of 1024 respondents (33.2%) attributed it to ‘insufficient opportunities’, 264 (25.8%) chose ‘discrimination’, 233 (22.8%) chose ‘lack of skillsets’, and 178 (17.4%) chose ‘lack of special employment agencies’.

### Needs and activities

Most respondents (75.4%) had joined patient organizations. As shown in Additional file 1: Fig. S1A, among these individuals, 1351 (94.1%) listed ‘communicate disease information’ as a major reason to join such organizations, and 1000 (69.6%) mentioned ‘obtain support and guidance from organizations’. Out of 1889 respondents, 693 (36.7%) rated the performance of patient organizations as ‘very good, but can be improved’, and 687 (36.4%) rated them as ‘extremely good’, indicating a general approval of the work of patient organizations. The 968 individuals who did not rate patient organizations as ‘extremely good’ were questioned on how to improve the work of patient organizations (Additional file 1: Fig. S1B). A total of 616 individuals (63.6%) suggested these organizations should ‘obtain more philanthropic support’, 614 (63.4%) suggested they could ‘provide more disease-related information and guidance’, 601 (62.1%) suggested ‘advocacy for legislations’, 579 (59.8%) suggested ‘raise rare disease awareness’, 424 (43.8%) suggested ‘protect patient rights’, and 267 (27.6%) suggested ‘maintain an equal and respectful environment for communication’.

The information needs of patients and care-givers were also assessed (Additional file 1: Fig. S1C). Out of 1923 respondents, 1538 (80.0%) selected information about ‘treatment’, 1429 (74.3%) selected ‘research progress’, 1282 (66.7%) selected ‘medical insurance’, and 1089 (56.6%) selected ‘contact information of experts’. Currently, most respondents (70.1%) obtained relevant information through social media, 1074 (54.8%) through physicians, 864 (44.1%) through websites, 675 (34.5%) through patient meetings. As to the preference of information source, 1419 out of 1911 respondents (74.3%) mentioned social media, 1334 (69.8%) selected physicians, and 1207 (63.2%) selected websites. There is clearly a discrepancy between information obtained and information the patient hoped to obtain from physicians.

Participants also made recommendations for policy-makers on how to help rare disease patients (Additional file 1: Fig. S1D). Out of 1924 respondents, 1725 (89.7%) recommended ‘support orphan drug development’, and 1681 (87.4%) recommended ‘appropriate medical insurance for rare disease patients’.

### Quality of life

A battery of standardized surveys was employed to assess the quality of life, health status, and psychological status of patients and care-givers. Firstly, results using the SF-36, patients and care-givers had remarkably lower scores in all domains than healthy populations (Table 2). With the PHQ-9, patients had an average score of 12.1 (95% CI 11.4–12.8), while care-givers had an average score of 9.5 (95%CI 9.1–9.9) (Table 3). The scores of patients and care-givers were higher than normative values and were in the category of moderate and mild depression, respectively. As to GAD-7, patients had an average score of 9.0 (95% CI 8.3–9.6), and care-givers had an average score of 7.7 (95% CI 7.4–8.0) (Table 3). Both patients and care-givers were in the category of (mild anxiety), and their average scores were significantly higher than normative values from the healthy population. With regard to PHQ-15, the average score of patients was 10.3 (95% CI 9.6–11.0), higher than the cut-off value of medium somatic symptom severity. The average score of care-givers was 8.1 (95% CI 7.6–8.5). The scores of patients and care-givers were markedly higher than normative values in healthy populations. Results from the PSQI showed that the average score of patients and care-givers were 8.5 (95% CI 8.0, 8.9) and 6.9 (95% CI 6.7, 7.2), respectively. These scores were also higher than normative values, indicating severe sleeping problems. There was a significant correlation between all seven domains of PSQI (Additional file 1: Table S1), and Cronbach’s alpha was 0.798, indicating a good internal consistency. Together, these results demonstrate that the disease burden led to poor health, anxiety, depression, somatization, and sleeping problems in patients and care-givers.

**Table 2:** Scores of patients and care-givers in SF-36

	Respondents				Healthy populations			
	Patients		Care-givers		Hubei [27]	Shanghai [28]	USA [29]	Canada [30]
	n = 259		n = 1061		n = 2249	n = 919	n = 2474	n = 9423
	Mean (SD)	95% CI	Mean (SD)	95% CI	Mean (SD)	Mean (SD)	Mean (SD)	Mean (SD)
PF	52.5 (34.8)	48.3, 56.6	70.2 (31.1)	68.4, 72.1	90.6 (15.4)	89.7 (14.8)	84.2 (23.3)	85.8 (20.0)
RP	26.8 (39.6)	21.0, 31.6	57.6 (46.0)	55.8, 60.3	79.5 (34.7)	92.8 (22.6)	81.0 (34.0)	82.1 (33.2)
BP	65.5 (32.0)	61.6, 69.3	82.7 (28.1)	81.0, 84.4	85.6 (18.4)	94.6 (13.8)	75.2 (23.7)	75.6 (23.0)
GH	32.6 (21.3)	30.0, 35.1	46.2 (22.7)	44.9, 47.6	69.6 (21.3)	68.8 (19.4)	72.0 (20.3)	77.0 (17.7)
VT	35.6 (22.5)	32.9, 38.3	46.2 (22.7)	44.9, 47.6	70.3 (17.1)	71.8 (18.3)	60.9 (21.0)	65.8 (18.0)
SF	33.3 (43.0)	28.2, 38.5	63.3 (25.1)	61.8, 64.8	86.9 (17.3)	94.3 (12.1)	83.3 (22.7)	86.2 (19.8)
RE	33.0 (42.8)	27.7, 38.2	55.8 (47.1)	53.0, 58.6	76.5 (38.5)	95.1 (20.6)	81.3 (33.0)	84.0 (31.7)
MH	41.4 (23.6)	38.6, 44.3	49.6 (22.1)	48.3, 50.9	72.7 (16.8)	81.8 (14.7)	74.7 (18.0)	77.5 (15.3)

**Table 3:** Scores of patients and care-givers in PHQ-9, GAD-7, PHQ-15, and PSQI

	Patients			Care-givers			Normative level		
	n	Mean (SD)	95% CI	n	Mean (SD)	95% CI	n	Mean (SD)	References
PHQ-9	305	12.1 (6.5)	11.4, 12.8	1258	9.5 (6.8)	9.1, 9.9	1003	3.3 (4.1)	[16]
GAD-7	304	9.0 (6.0)	8.3, 9.6	1256	7.7 (5.9)	7.4, 8.0	5030	3.0 (3.4)	[10]
PHQ-15	290	10.3 (6.1)	9.6, 11.0	1194	8.1 (6.5)	7.6, 8.5	9250	5.5 (3.9)	[31]
							5031	3.8 (4.1)	[32]
PSQI	286	8.5 (3.9)	8.0, 8.9	1184	6.9 (4.0)	6.7, 7.2	9284	5.0 (3.4)	[33]
							629	4.9 (2.4)	[13]

Furthermore, correlations between PHQ-9, PHQ-15, GAD-7, PSQI, and subscales of SF-36 were analyzed (Additional file 1: Table S2). Moreover, the impact of age, disease types, and gender on scores in these tests were analyzed by a general linear model. Relationship (patients or care-givers) only had an impact on the scores of PHQ-15, PSQI, and GH of SF-36. Specifically, patients had a higher score in PHQ-15 than care-givers (difference = 2.541, adjusted *p* = 0.004), indicating more severe somatic symptoms. Patients had a higher score in PSQI than care-givers (difference = 1.829, adjusted *p* = 0.002), indicating more severe sleeping problems in patients. Patients had a significantly lower score in GH of SF-36 than care-givers (difference = − 11.327, adjusted *p* = 0.001), indicating that the disease burden significantly affected the general health of patients. Disease had an impact on the scores of PHQ-9, PHQ-15, GAD-7, PSQI, and subscales of SF-36 (Additional file 1: Table S3). Generally speaking, poor health, anxiety, depression, somatization, and sleeping problems were more severe in patients and care-givers of more severe diseases (e.g., DMD, hemophilia, Dravet) or undiagnosed.

## Discussion

Delayed diagnosis and misdiagnosis is common and can lead to delayed and improper treatment, as well as accelerating the deterioration of the disease [29]. As a result, unnecessary tests and treatments are often conducted, resulting in significant cost to patients and the healthcare system [19]. A previous study in 2013 reported that it took 5–7 years and 2–3 misdiagnoses in the USA and UK [30]. Interestingly, the duration between onset and diagnosis in China was 1.4 years, and there were only 1.8 misdiagnoses. This may be because of the increased application of newborn screening over the years in China [31]. It may be also because social media has increased avenues for relevant information and communication between patients.

One major reason for the difficulty in diagnosis is the limited rare disease awareness among patients, families, and physicians. Similar to a previous study in Europe [32], as believed by patients and care-givers in this study, physicians did not have sufficient awareness and knowledge about rare diseases. A recent study among physicians in China also showed a lack of rare disease awareness [33]. Therefore, there is a critical need to include rare disease content in medical education and training. Further, a Chinese information hub of rare diseases was suggested to provide reliable, up-to-date, and ‘peer-reviewed’ information and guidance.

As shown in this study, many patients were facing various challenges in life, education and employment. Firstly, 725 (39.8%) of 1830 respondents found no available barrier-free facilities in public places, and 59.7% of patients had confronted difficulties in education. The lack of barrier-free facilities may result from the insufficient attention and awareness to rare diseases in public, while difficulties in education may due to deficient equipment or staffs from public organizations and departments to help the disabled to learn. Worse, most of respondents suffered great financial hardship. Compared with employment status in Europe that 61% of rare disease patients and care-givers had experienced interruptions in their professional activities [34], this situation in China seemed to be even more severe. Only 176 (9.4%) of 1881 patients had their own jobs and 69.6% of respondents relied on the earning of their family members. From the above results, there existed employment discriminations towards rare disease patients, which was not only on account of the patients’ poor health, but also due to the lack of employment opportunity and assistances from the government.

Additionally, this study also suggested that the majority of patients and care-givers were most interested in information about treatment. Orphan drug development is always expensive and time-consuming, ultimately affecting patients’ access to new treatments. Witnessing the success of orphan drug acts in other countries [35, 36], there was high support for an orphan drug act in China. Another relevant factor is the low medical insurance coverage, significantly affecting the viability of orphan drugs in the Chinese market. This not only affects the orphan drug development in China, but also affects the import of orphan drugs from abroad.

Based on the aforementioned results and discussions which reflected the stress and need of most rare disease patients, our team, in the perspective of investigators, proposed some key recommendations that outline top priorities for policymakers in China to consider, and suggestions are listed as follows:

1. Pass an Orphan Drug Act to stimulate orphan drug development. Learn from Orphan Drug Act of America, some effective policies should be included [37]. Firstly, provide government funding for clinical research of orphan drug. Secondly, reduce requirements for clinical trials of orphan drug. Thirdly, the period of monopoly protection for orphan drugs should be reasonably extended. Fourthly, increase tax deductions and exemptions. Last but not least, carry out rapid approval and priority approval for the approval process of orphan drugs. Apart from proposing a better bill, the decision makers should clearly know the difficult status quo of patients with rare diseases and the urgent need for Orphan Drug Act to stimulate orphan drug development.
2. Official definition or suggested threshold of rare diseases. To propose an official definition of rare diseases in China, many factors should be taken into consideration such as epidemiology, population, economics, social medical security system and patients' living conditions. Besides, referring to the definitions for rare diseases of other countries and regions is necessary, for instance the threshold for rare diseases in EU is below 0.5‰ [1] while Taiwan is below 0.1‰ [3].
3. Improve rare disease awareness among medical professionals and the general public. For medical professionals, the government, profit organization as well as non-profit organizations should arrange rare disease knowledge popularization and interchange courses regularly by the assistance of rare disease experts. And online or face-to-face communication meeting for doctors and rare disease patients ought to be held monthly or quarterly, which could help medical professionals better understand the conditions of patients. For general public, more articles and videos about rare diseases should be published and broadcasted on the online official accounts, some courses about rare diseases ought to be included as the compulsory courses for knowledge popularization in schools. Considering the celebrity effect, some celebrities can be invited to appeal public to care about and help patients with rare diseases. What's more, a Chinese information hub of rare diseases was suggested to provide reliable, up-to-date, and ‘peer-reviewed’ information and guidance.
4. Improve the medical and commercial insurance coverage of treatments and care for rare diseases. National Essential Medicines List should include more drugs for rare diseases. Basic medical insurance should extensively cover more drugs for rare diseases, and their medical insurance reimbursement ratio ought to be increased. Starting from March 1, 2021, China fully implemented the national medical insurance directory for 2020, with seven rare disease treatment drugs included in the national medical insurance directory [38]. There is no doubt that this policy can better ease the financial burden of related patients and families with rare disease, and we hope more similar policies to show up in future. Additionally, promote the establishment of commercial insurance for rare diseases and make a clear regulation that the insurance company cannot refuse the insurance of rare diseases. The government can encourage the commercial insurance institutions to participate in the provision of serious disease and rare disease insurance by the way of granting tax exemptions and business tax exemptions for insurance business supervision fees and insurance guarantee funds.
5. Improve the availability and affordability of prenatal screening, newborn screening, and genetic counseling. Increase more free prenatal diagnosis and neonatal screening programs, such as the free prenatal screening for Down Syndrome nationally, the free newborn screening for Glucose-6-phosphatase deficiency nationally, and cheaper genetic counseling for all parents. Certainly, all of the free programs and cheaper counseling must be firmly supported by the government’s policies and fund subsidy.
6. Prevent discrimination against rare disease patients in education and employment. Establish more special schools or classes for rare disease patients online to offline regionally, and recruit some professional teachers to teach them some survival skills and life knowledge. The government and employment institution should provide more opportunities for rare disease patients, for example Alibaba has provided many online customer service positions for brittle bone disease patients since 2011, which could ease their economic stress in some degree and have set a good example for other companies [39].
7. Accelerate in importing orphan drugs. Actually, the government have published that only 3% import value-added tax need to be paid without import tariff since March 1, 2019 when importing 21 kinds of orphan drugs [40]. There is no doubt that this policy could promote the import of some orphan drugs effectively. To be frank, the kinds of orphan drugs should be increased in this policy list, and simplify the approval process for the import of orphan drugs already on the market abroad, both of which can help to accelerate the import of orphan drugs.

Sincerely, we hope above commendations could be adopted by the policymakers in China to drive the improvement of status quo of rare disease communities. When it comes to this special group with rare diseases, we ever meditated whether 'individual' instead of 'patient' is more appropriate to describe them, because the word 'individual' shows better respect and care from the public to them. And the word 'individual' also reflects better equality among rare disease group and other populations than 'patient'. However, on account of the lacking understanding about the special group with rare diseases, most of people regard them as the population with illness at present. Maybe the word 'patient' is more in line with the current people's cognition than 'individual' when we mention the group with rare diseases. In conclusion, we choose the word 'patient' to introduce them, while we strongly hope ‘individual’ rather than ‘patient’ could be broadly used to describe the group with rare diseases in future after our efforts to raise the public awareness of rare diseases. All the better changes need our joint efforts.

### Limitations

This online survey was invited to patients or care-givers (age 18 or above) through social media. Given the legal age of the adult in China is 18, which indicates they are the individuals with full capacity for civil conduct and can think independently as well as earn their livings, the potential participants of the questionnaire were the adults. The adults can better understand the questions and better know about their own health and financial stress than minors, so that the results could be more true and valid. Nevertheless, some actual medical content could not be verified by our quality criteria. It is assumed that the high percent of female participants to the study is over-representative, which may bias the survey findings. This may because most care-givers are women, and they are more likely to respond to surveys.

Besides, there are 904 million (64.5%) internet users in China by March 2020, but some patients and care-givers from remote mountainous region still could not be invited to complete this survey because of underdeveloped network, leading to biased evaluation of status quo of rare diseases to some extent. As this research is an online survey, these survey data and results may not be representative for the whole population of rare disease patients and caregivers in China. The questionnaire was initially released on the “Zhihu” website and Seven Pansy Rare Diseases Platform, only those users who arrived at our page and saw our questionnaire link, who were willing to click and enter the link to answer the questionnaire are our real interviewees, which could lead to incomprehensive coverage about the current situation of patients with rare diseases and their care-givers in China.

Last but not least, as we showed above, the HRQOL indicators in rare disease questionnaire could help to better know the life quality of rare disease patients, but it still had limitations. Actually, the clinical picture and prognosis of various rare diseases are different, so mixing results of all rare diseases using HRQOL indicators together could lead to decline in accuracy of specific rare diseases’ real data and reduced significance to draw some conclusions as well as key suggestions in some degree.

In brief, there did exist some limitations in this questionnaire design as well as manuscript, so our team will struggle for a better survey design to get more comprehensive research results in future. We hope these data and conclusions could be published to successfully attract enough attention on the unamiable status quo of rare disease patients in China, and drive all social strata to make efforts to improve the life quality of this special group.

## Conclusions

This is the first large-scale study that quantitatively investigates the current status of rare disease patients in China. This study identifies poor health status, depression, somatization, anxiety, and sleeping issues among both patients and care-givers through standardized test, highlighting the need of psychological and medical support. Also, this study also observes difficulties in social life, education, and employment, as well as financial burden. More importantly, the most urgent and critical need of these patients are treatment/orphan drugs. Therefore, a list of action items is recommended to the policy-makers, which include legislating an orphan drug act, raising rare disease awareness, and preventing discrimination.

## Supplementary Information

**Additional file 1.** **Table S1.** Correlation between scores of PHQ-9, GAD-7, PHQ-15, PSQI, and 8 domains of SF-36. **Table S2.** Correlation between scores of PSQI and its 7 domains. **Table S3.** The impact of disease on scores of PHQ-9, GAD-7, PSQI, PHQ-15, and SF-36. **Fig. S1.** Attitude of patients and care-givers towards patient organizations, information needs, and government.

## Data Availability

The raw data are not publicly available due to the agreement between the investigators and the participants.

## References

[1] Bax BE (2021). Biomarkers in rare diseases. Int J Mol Sci.

[2] Gong S (2016). The availability and affordability of orphan drugs for rare diseases in China. Orphanet J Rare Dis.

[3] Chu SY, Weng CY (2017). Introduction to genetic/rare disease and the application of genetic counseling. Hu li za zhi J Nurs.

[4] Pariser AR, Gahl WA (2014). Important role of translational science in rare disease innovation, discovery, and drug development. J Gen Intern Med.

[5] Rare diseases—World Health Organization. https://www.who.int/medicines/areas/priority_medicines/Ch6_19Rare.pdf?ua=1. Accessed 10 Aug 2020.

[6] Rajasimha HK (2014). Organization for rare diseases India (ORDI)—addressing the challenges and opportunities for the Indian rare diseases' community. Genet Res.

[7] Melnikova I (2012). Rare diseases and orphan drugs. Nat Rev Drug Discov.

[8] Global Genes. RARE facts. https://globalgenes.org/rare-facts/. Accessed 10 Aug 2020.

[9] Schadewald A, Kimball E, Ou L (2018). Coping strategies, stress, and support needs in caregivers of children with mucopolysaccharidosis. JIMD Rep.

[10] Song P, He J, Li F, Jin C (2017). Innovative measures to combat rare diseases in China: the national rare diseases registry system, larger-scale clinical cohort studies, and studies in combination with precision medicine research. Intractable Rare Dis Res.

[11] He J, Kang Q, Hu J, Song P, Jin C (2018). China has officially released its first national list of rare diseases. Intractable Rare Dis Res.

[12] He J (2019). Incidence and prevalence of 121 rare diseases in China: current status and challenges. Intractable Rare Dis Res.

[13] Ren Q, Wang J (2019). Network established to collaborate on diagnosis and treatment of rare diseases in China: a strategic alliance backed by tiered healthcare is the key to the future. Intractable Rare Dis Res.

[14] Tao W (2020). Towards universal health coverage: lessons from 10 years of healthcare reform in China. BMJ Glob Health.

[15] He J (2018). China issues the National Essential Medicines List (2018 edition): background, differences from previous editions, and potential issues. Biosci Trends.

[16] Lv M, Chang F (2017). Analysis on problems and countermeasures of medical insurance for rare diseases in China under the international environment. Chin J Med Guide.

[17] HRQOL Concept. https://www.cdc.gov/hrqol/concept.htm. Accessed 25 May 2021.

[18] Ware JE, Sherbourne CD (1992). The MOS 36-item short-form health survey (SF-36). I. Conceptual framework and item selection. Med Care.

[19] Buysse DJ, Reynolds CF, Monk TH, Berman SR, Kupfer DJ (1989). The Pittsburgh sleep quality index: a new instrument for psychiatric practice and research. Psychiatry Res.

[20] Löwe B (2008). Validation and standardization of the generalized anxiety disorder screener (GAD-7) in the general population. Med Care.

[21] Kroenke K, Spitzer RL, Williams JB (2001). The PHQ-9: validity of a brief depression severity measure. J Gen Intern Med.

[22] Spitzer RL, Kroenke K, Williams JB, Löwe B (2006). A brief measure for assessing generalized anxiety disorder: the GAD-7. Arch Intern Med.

[23] Guo S, Sun W, Liu C, Wu S (2016). Structural validity of the Pittsburgh sleep quality index in Chinese undergraduate students. Front Psychol.

[24] Zhang Y, Qu B, Lun SS, Guo Y, Liu J (2012). The 36-item short form health survey: reliability and validity in Chinese medical students. Int J Med Sci.

[25] Li L, Wang HM, Shen Y (2003). Chinese SF-36 health survey: translation, cultural adaptation, validation, and normalisation. J Epidemiol Community Health.

[26] Wang W (2014). Reliability and validity of the Chinese version of the patient health questionnaire (PHQ-9) in the general population. Gen Hosp Psychiatry.

[27] Tong X, An D, McGonigal A, Park SP, Zhou D (2016). Validation of the generalized anxiety disorder-7 (GAD-7) among Chinese people with epilepsy. Epilepsy Res.

[28] Forman J (2012). The need for worldwide policy and action plans for rare diseases. Acta paediatr (Oslo, Norway: 1992).

[29] Communication from the Commission to the European Parliament. https://ec.europa.eu/health/ph_threats/non_com/docs/rare_com_en.pdf. Accessed 10 Aug 2020.

[30] Rare Disease Impact Report. https://globalgenes.org/wp-content/uploads/2013/04/ShireReport-1.pdf. Accessed 10 Aug 2020.

[31] Li X (2020). The urgent need to empower rare disease organizations in China: an interview-based study. Orphanet J Rare Dis.

[32] Estudio ENSERio. https://enfermedades-raras.org/images/stories/documentos/Estudio_ENSERio.pdf. Accessed 10 Aug 2020.

[33] Li X, et al. Rare disease awareness and perspectives of physicians in China: a questionnaire-based study. Orphanet J Rare Dis. 2021;16(1):171. 10.1186/s13023-021-01788-3. 10.1186/s13023-021-01788-3 PMC8042908 33849615

[34] The Voice of 12,000 Patients. https://www.eurordis.org/IMG/pdf/voice_12000_patients/EURORDISCARE_FULLBOOKr.pdf. Accessed 10 Aug 2020.

[35] Orphan Drug Act of 1983. https://www.govinfo.gov/content/pkg/STATUTE-96/pdf/STATUTE-96-Pg2049.pdf. Accessed 10 Aug 2020*.*

[36] Groft SC (2013). Rare diseases research: expanding collaborative translational research opportunities. Chest.

[37] Gabay M (2019). The Orphan drug act: An appropriate approval pathway for treatments of rare diseases?. Hosp Pharmacy.

[38] The National Health Insurance Directory 2020 will be fully implemented tomorrow, including seven rare disease treatments. https://baijiahao.baidu.com/s?id=1692926975621732588&wfr=spider&for=pc. Accessed 25 May 2021.

[39] Hundreds of patients with rare diseases in China have found cloud jobs on Alibaba. http://roll.sohu.com/20150808/n418427959.shtml. Accessed 25 May 2021.

[40] The new policy of preferential VAT tax was implemented for 21 drugs for rare diseases. https://www.sohu.com/a/299996336_120036865. Accessed 25 May 2021.

