# Supplementary material for: A questionnaire-based study to comprehensively assess the status quo of rare disease patients and care-givers in China
Source: Orphanet J Rare Dis. 2021 Jul 22;16:327. doi: 10.1186/s13023-021-01954-7 (PMC8296703; doi:10.1186/s13023-021-01954-7)
Supplement: Supplementary file 1 — Additional file 1. Table S1. Correlation between scores of PHQ-9, GAD-7, PHQ-15, PSQI, and 8 domains of SF-36. Table S2. Correlation between scores of PSQI and its 7 domains. Table S3. The impact of disease on scores of PHQ-9, GAD-7, PSQI, PHQ-15, and SF-36. Fig. S1. Attitude of patients and care-givers towards patient organizations, information needs, and government. [file 13023_2021_1954_MOESM1_ESM.docx]

|  | **PF** | **RP** | **BP** | **GH** | **VT** | **SF** | **RE** | **MH** | **PHQ9** | **GAD7** | **PHQ15** | **PSQI** |
| --- | --- | --- | --- | --- | --- | --- | --- | --- | --- | --- | --- | --- |
| **PF** |  | .623** | .628** | .587** | .381** | .500** | .454** | .296** | -.335** | -.300** | -.316** | -.305** |
| **RP** |  |  | .574** | .541** | .415** | .521** | .662** | .374** | -.357** | -.323** | -.336** | -.316** |
| **BP** |  |  |  | .610** | .484** | .572** | .525** | .408** | -.461** | -.419** | -.501** | -.476** |
| **GH** |  |  |  |  | .596** | .565** | .471** | .507** | -.414** | -.382** | -.347** | -.347** |
| **VT** |  |  |  |  |  | .526** | .512** | .768** | -.667** | -.630** | -.542** | -.580** |
| **SF** |  |  |  |  |  |  | .578** | .528** | -.546** | -.533** | -.407** | -.437** |
| **RE** |  |  |  |  |  |  |  | .522** | -.477** | -.453** | -.384** | -.410** |
| **MH** |  |  |  |  |  |  |  |  | -.698** | -.706** | -.505** | -.566** |
| **PHQ9** |  |  |  |  |  |  |  |  |  | .846** | .611** | .590** |
| **GAD7** |  |  |  |  |  |  |  |  |  |  | .588** | .576** |
| **PHQ15** |  |  |  |  |  |  |  |  |  |  |  | .631** |
| **PSQI** |  |  |  |  |  |  |  |  |  |  |  |  |

**Table S1. Correlation between scores of PHQ-9, GAD-7, PHQ-15, PSQI, and 8 domains of SF-36.** Pearson correlation, **, p< 0.01 (2-tailed).

|  | **SQ** | **SL** | **SD** | **SE** | **Dis** | **SM** | **DD** | **PSQI** |
| --- | --- | --- | --- | --- | --- | --- | --- | --- |
| **SQ** |  | .571** | .485** | .315** | .568** | .279** | .595** | .813** |
| **SL** |  |  | .331** | .255** | .509** | .177** | .449** | .728** |
| **SD** |  |  |  | .498** | .377** | .159** | .400** | .697** |
| **SE** |  |  |  |  | .217** | .131** | .187** | .561** |
| **Dis** |  |  |  |  |  | .216** | .585** | .737** |
| **SM** |  |  |  |  |  |  | .189** | .393** |
| **DD** |  |  |  |  |  |  |  | .744** |
| **PSQI** |  |  |  |  |  |  |  |  |

**Table S2. Correlation between scores of PSQI and its 7 domains.** Pearson correlation, **, p< 0.01 (2-tailed).

| **PHQ-9** | CAH<NF (-5.543, p=0.004), undiagnosed (-4.493, p=0.002) |
| --- | --- |
| **GAD-7** | CAH<Dravet (-6.696, p=0.048), NF (-5.508, p<0.0001), undiagnosed (-4.798, p<0.0001) |
|  | Hemophilia<NF (-4.527, p=0.031) |
|  | PKU<NF (-5.760, p=0.016), undiagnosed (-2.109, p=0.049) |
|  | Other<undiagnosed (-5.050, p=0.031) |
| **PSQI** | CAH<NF (-2.553, p=0.014) |
| **PF** | CAH>DMD (55.130, p<0.0001), hemophilia (30.266, p=0.002), MPS (53.510, p<0.0001), other (34.438, p<0.0001) |
|  | DMD<hemophilia (-24.864, p=0.012), neurofibromatosis (-45.953, p<0.0001), PKU (-34.858, p=0.001), undiagnosed (-42.687, p<0.0001), other (-20.693, p=0.001) |
|  | MPS<NF (-44.311, p=0.001), undiagnosed (-41.067, p=0.001) |
|  | Other<NF (-25.239, p<0.0001), undiagnosed (-21.994, p<0.0001) |
| **RP** | CAH>DMD (51.131, p<0.0001), Dravet (53.093, p=0.041), hemophilia (62.844, p<0.0001), MPS (61.948, p=0.006), other (47.042, p<0.0001), undiagnosed (40.342, p<0.0001) |
|  | Hemophilia<NF (-41.802, p=0.002), PKU (-47.094, p=0.011) |
|  | Other<NF (-26.000, p=0.013) |
| **BP** | CAH>DMD (29.047, p<0.0001), hemophilia (36.521, p<0.0001), other (21.564, p<0.0001), and undiagnosed (16.765, p=0.045) |
|  | Hemophilia<NF (-25.195, p=0.003), PKU (-29.004, p=0.021), undiagnosed (-19.756, p=0.020) |
| **SF** | CAH>DMD (24.621, p<0.0001), Dravet (32.447, p=0.008), hemophilia (20.627, p=0.015), other (16.835, p=0.002), undiagnosed (20.965, p<0.0001) |
|  | PKU>DMD (24.513, p=0.015), Dravet (32.338, p=0.034), undiagnosed (20.857, p=0.028) |
| **RE** | CAH>DMD (43.151, p=0.001), Dravet (62.577, p=0.007), hemophilia (44.432, p=0.003), other (45.237, p<0.0001), undiagnosed (42.651, p<0.0001) |
| **MH** | CAH>NF (17.980, p=0.011), undiagnosed (13.928, p=0.036) |
|  | Hemophilia>NF (18.401, p=0.019) |

**Table S3. The impact of disease on scores of PHQ-9, GAD-7, PSQI, PHQ-15, and SF-36.**

Since there were 104 types of diseases, to simplify the analysis, diseases were categorized into PKU, MPS, DMD, neurofibromatosis (NF), Dravet, CAH, undiagnosed, and other. Data were represented as mean difference and adjusted p value. Only comparisons with statistical significance were shown.

**Fig. S1. Attitude of patients and care-givers towards patient organizations, information needs, and government. (A)** The motivation of patients and care-givers to join a certain patient organization. **(B)** Recommendations to patient organizations on how to improve performance. **(C)** Current information source and preferred information source. **(D)** Recommendations to government.
